# ID 124 - Dossiê de Valor 2024: facilitando a compreensão dos indicadores de saúde para pacientes

**DOI:** 10.5327/2237-9622.2025.v34s1.124

**Published:** 2025-11-25

**Authors:** Daniel Tavares Malheiro, Sabrina Bernardes Pereira, Fabio Andre Nanci Izidro Goncalves, Daisa de Masquita Escobosa

**Keywords:** ATS, valor em saúde, indicadores

## Abstract

**Introdução:**

A comunicação eficaz em saúde é essencial para capacitar os pacientes a tomarem decisões informadas sobre seu tratamento. Informações transparentes e acessíveis podem melhorar a compreensão dos pacientes sobre suas condições e opções terapêuticas, impactando positivamente a adesão ao tratamento e os resultados de saúde.

**Método:**

Para construção do material foi feita inicialmente uma revisão de literatura a fim de identificar formas de comunicação de indicadores de saúde para o público leigo. Baseado no modelo de por procedimento cirúrgico do Hospital for Special Surgery. Foi elaborado um protótipo de por condição de saúde com o conteúdo dos indicadores publicados no Dossiê de Valor 2024. O protótipo foi discutido e revisado pelos times de Experiência do Paciente, Comunicação e Jurídico. O material final foi revisado por um conselho editorial.

**Resultados:**

O material foi lançado no Fórum de Qualidade e Segurança do IHI em julho de 2024, abrangendo 20 condições de saúde, com uma média de cinco indicadores por condição clínica. Disponibilizado para o corpo clínico e no site do Einstein por meio do seguinte link:

Dossiê de Valor 2024 – Versão Pacientes

A versão simplificada apresenta dados de forma clara e compreensível, promovendo maior transparência e facilitando o acesso dos pacientes a informações essenciais para decisões informadas sobre seus tratamentos.

**Conclusão:**

A transparência em saúde é fundamental para assegurar que os pacientes tenham acesso a informações claras e precisas, permitindo-lhes tomar decisões informadas e fundamentadas sobre seu tratamento. Ao proporcionar acesso a dados detalhados e compreensíveis, o Dossiê de Valor Einstein fortalece a capacidade dos pacientes de participar ativamente do seu cuidado, promovendo escolhas mais fundamentadas e alinhadas com suas necessidades e preferências.

